# Combined Assessment of D-Dimer with the Get with the Guidelines—Heart Failure Risk Score and N-Terminal Pro-B-Type Natriuretic Peptide in Patients with Acute Decompensated Heart Failure with Preserved and Reduced Ejection Fraction

**DOI:** 10.3390/jcm10163564

**Published:** 2021-08-13

**Authors:** Hiroyuki Naruse, Junnichi Ishii, Hiroshi Takahashi, Fumihiko Kitagawa, Eirin Sakaguchi, Hideto Nishimura, Hideki Kawai, Takashi Muramatsu, Masahide Harada, Akira Yamada, Wakaya Fujiwara, Mutsuharu Hayashi, Sadako Motoyama, Masayoshi Sarai, Eiichi Watanabe, Hiroyasu Ito, Yukio Ozaki, Hideo Izawa

**Affiliations:** 1 Faculty of Medical Technology, School of Health Sciences, Fujita Health University, Toyoake 470-1192, Japan; 2 Department of Joint Research Laboratory of Clinical Medicine, Bantane Hospital, Nagoya 454-8509, Japan; jishii@fujita-hu.ac.jp; 3 Division of Statistics, Fujita Health University School of Medicine, Toyoake 470-1192, Japan; hirotaka@fujita-hu.ac.jp; 4 Department of Cardiology, Okazaki Medical Center, Fujita Health University School of Medicine, Okazaki 444-0827, Japan; fkitaga@fujita-hu.ac.jp (F.K.); sakaguch@fujita-hu.ac.jp (E.S.); ozakiyuk@fujita-hu.ac.jp (Y.O.); 5 Department of Cardiology, Fujita Health University School of Medicine, Toyoake 470-1192, Japan; hidetoishmura0621@gmail.com (H.N.); hidekikawai@xc4.so-net.ne.jp (H.K.); takam@fujita-hu.ac.jp (T.M.); mharada@fujita-hu.ac.jp (M.H.); a-yamada@fujita-hu.ac.jp (A.Y.); muhayasi@med.nagoya-u.ac.jp (M.H.); sadakom@fujita-hu.ac.jp (S.M.); msarai@fujita-hu.ac.jp (M.S.); izawa@fujita-hu.ac.jp (H.I.); 6 Department of Cardiology, Bantane Hospital, Nagoya 454-8509, Japan; wakayafj@fujita-hu.ac.jp (W.F.); enwatan@fujita-hu.ac.jp (E.W.); 7 Department of Joint Research Laboratory of Clinical Medicine, Fujita Health University School of Medicine, Toyoake 470-1192, Japan; hiroyasu.ito@fujita-hu.ac.jp

**Keywords:** D-dimer, GWTG-HF risk score, NT-proBNP, HFpEF, HFrEF, risk stratification

## Abstract

The prognostic role of D-dimer in different types of heart failure (HF) is poorly understood. We investigated the prognostic value of D-dimer on admission, both independently and in combination with the Get With The Guidelines—Heart Failure (GWTG-HF) risk score and N-terminal pro-B-type natriuretic peptide (NT-proBNP), in patients with preserved left ventricular ejection fraction (LVEF) and acute decompensated HF (HFpEF) or reduced LVEF (HFrEF). Baseline D-dimer levels were measured on admission in 1670 patients (mean age: 75 years) who were hospitalized for worsening HF. Of those patients, 586 (35%) were categorized as HFpEF (LVEF ≥ 50%) and 1084 as HFrEF (LVEF < 50%). During the 12-month follow-up period after admission, 360 patients died. Elevated levels (at least the highest tertile value) of D-dimer, GWTG-HF risk score, and NT-proBNP were all independently associated with mortality in all HFpEF and HFrEF patients (all *p* < 0.05). Adding D-dimer to a baseline model with a GWTG-HF risk score and NT-proBNP improved the net reclassification and integrated discrimination improvement for mortality greater than the baseline model alone in all populations (all *p* < 0.001). The number of elevations in D-dimer, GWTG-HF risk score, and NT-proBNP were independently associated with a higher risk of mortality in all study populations (HFpEF and HFrEF patients; all *p* < 0.001). The combination of D-dimer, which is independently predictive of mortality, with the GWTG-HF risk score and NT-proBNP could improve early prediction of 12-month mortality in patients with acute decompensated HF, regardless of the HF phenotype.

## 1. Introduction

D-dimer is the end product of plasmin-mediated degradation of cross-linked fibrin. Plasma concentrations of D-dimer, a marker of coagulation, are dependent on fibrin generation and subsequent degradation by the endogenous fibrinolytic system [1]. Patients with heart failure (HF) have been associated with hemostatic abnormalities [2,3]. Several studies have suggested that elevated D-dimer levels are associated with adverse outcomes in patients hospitalized for HF [4,5,6,7]. HF with preserved ejection fraction (HFpEF) and HF with reduced ejection fraction (HFrEF) are two distinct HF phenotypes with different etiologic factors and pathophysiologic mechanisms [8]; however, they share similar rates of mortality [9,10,11]. The prognostic role of D-dimer in different types of HF, particularly in HFpEF patients, is poorly understood.

The Get With The Guidelines—Heart Failure (GWTG-HF) risk score was established to predict in-hospital mortality in patients with acute HF [12]. Recent studies demonstrated that the GWTG-HF risk score could provide prognostic information in not only the acute phase during HF hospitalization, but also during the chronic phase following HF discharge [13,14]. However, due to the complexity of HF, it is difficult to establish reliable risk stratification for adverse outcomes based on only demographic and clinical information, and routine laboratory findings [15]. Serum N-terminal pro-B-type natriuretic peptide (NT-proBNP) levels have been widely used and are an established biomarker for predicting prognosis in patients with HF [16,17]. Previous studies have shown improved predictions of adverse outcomes following the addition of natriuretic peptides to the GWTG-HF risk score in patients with acute HF [18,19,20,21].

In this study, we assessed the prognostic value of D-dimer levels both independently and in combination with the GWTG-HF risk score and NT-proBNP levels, for acute decompensated HF patients with HFpEF and HFrEF.

## 2. Materials and Methods

### 2.1. Study Design

This study was conducted at the Department of Cardiology, Fujita Health University School of Medicine (Toyoake, Japan). The ethics committee of Fujita Health University approved this study (study protocol number 13-119), which was performed in accordance with the Declaration of Helsinki. All patients individually provided written informed consent.

Patients hospitalized for worsening HF at medical (non-surgical) cardiac intensive care units (CICU) from January 2013 to December 2019 were enrolled in this investigation. Upon admission, blood samples were obtained to determine baseline biomarker measurements. Patients who had the following characteristics were excluded from participation: (1) under the age of 18 years old, (2) currently undergoing cardiac surgery, (3) the presence of stage five chronic kidney disease, (4) clinical or electrocardiographic evidence suggestive of an acute coronary syndrome in the 3 months preceding admission, (5) a previous diagnosis of confirmed deep vein thrombosis and/or pulmonary thromboembolism, (6) a previous diagnosis of acute aortic dissection, (7) receiving percutaneous cardiopulmonary support before admission, (8) having an active malignant disease being treated with chemotherapy or radiation, (9) having autoimmune diseases, and (10) experiencing trauma.

Physicians independently selected the appropriate therapy and managed the patients following standard protocols using outcome measurements such as an improvement in symptoms, physical examination findings, pulmonary congestion on a chest radiograph, and echocardiographic findings. Clinical characteristics were obtained from patients’ medical records upon enrollment.

### 2.2. Definitions and Calculations

All patients were subjected to M-mode and two-dimensional echocardiography at the time of enrollment. All scans were conducted by experts blinded to the study details. Left ventricular ejection fraction (LVEF) was classified according to the baseline data. Specifically, HFpEF was defined as LVEF ≥ 50% and HFrEF as LVEF < 50% [22]. Diabetes was defined as having a history of or current diabetes, a fasting plasma glucose level of ≥126 mg/dL, a hemoglobin A1c value of ≥6.5%, or the presence of diabetic retinopathy. Hypertension was defined as having a systolic blood pressure of ≥140 mmHg, a diastolic blood pressure of ≥90 mmHg, or a history of antihypertensive treatment. Dyslipidemia was defined as a total cholesterol level of ≥220 mg/dL or a history of lipid-lowering therapy. Patients with a smoking history were classified as either current or ex-smokers.

The GWTG-HF risk score was calculated using the seven variables as previously reported [12]. The GWTG-HF risk score was calculated during admission and was based on race, age, systolic blood pressure, heart rate, blood urea nitrogen level, sodium concentration, and presence of chronic obstructive pulmonary disease. All patient scores were obtained by summing points assigned to the value of each predictor. The values of the score were between 0 and 100.

### 2.3. Outcomes

All patients were clinically followed up for 12 months after study enrollment. The primary endpoint, which was judged independently by researchers, was all-cause and cardiovascular mortality. Data for the endpoints were obtained from hospital charts and through telephone interviews with patients. Telephone interviews were conducted by trained reviewers who were blinded to the study details.

### 2.4. Measurement of Biochemical Markers

For the baseline measurement of plasma D-dimer and serum NT-proBNP, blood samples were collected and centrifuged at 1000× *g* at 4 °C for 15 min to isolate plasma. Plasma was then separated and stored at −80 °C until analysis. Plasma D-dimer levels were measured with a latex-enhanced photometric immunoassay (LPIA-ACE D-Dimer, LSI Medience Corporation, Tokyo, Japan). Serum NT-proBNP was measured using an electrochemiluminescence immunoassay and a Cobas e601 system (Roche Diagnostics, Tokyo, Japan). Serum high-sensitivity C-reactive protein (hs-CRP) levels were measured using a latex-enhanced hsCRP immunoassay (N-Latex CRP II, Siemens Healthineers Japan, Tokyo, Japan). Serum high-sensitivity troponin I (hs-TnI) levels were measured via chemiluminescence immunoassays using an ARCHITECT i2000SR system (Abbott Japan Co., Ltd., Tokyo, Japan).

### 2.5. Statistical Analyses

All statistical analyses were performed using StatFlex version 6 (Artech Co. Ltd., Osaka, Japan). Normally distributed variables are expressed as mean values ± standard deviations, whereas nonparametric data are presented as medians and interquartile ranges. Plasma D-dimer, serum NT-proBNP, serum hs-TnI, and serum hs-CRP data were non-normally distributed, so analyses were performed after log-transformation to meet the criteria for use in normalized statistical approaches (after statistical confirmation).

Intergroup differences were evaluated by one-way analysis of variance or the Kruskal-Wallis test for continuous variables and by the chi-square test for categorical variables. We examined the intergroup differences in endpoint data using the Kaplan-Meier method and compared the results using a log-rank test. Hazard ratios and 95% confidence intervals were calculated for each factor using the Cox proportional hazards analysis. All baseline variables with *p* < 0.05 in univariate analyses were integrated into the Cox multivariate model to determine the independent predictors of endpoints.

To assess whether the accuracy of predicting endpoints would improve after adding D-dimer into a baseline model with GWTG-HF risk score and NT-proBNP, we calculated the C-index, net reclassification improvement (NRI), and integrated discrimination improvement (IDI). The C-index was defined as the area under the receiver operating characteristic curves between individual predictive probabilities for endpoints and the incidence of endpoints, and it was compared with the baseline model [23]. NRI was a relative indicator of how many patients had improved in the predicted probability of endpoints. In contrast, IDI indicated the average improvement in the predicted probability of endpoints after adding variables to the baseline model [24]. A *p*-value of <0.05 was considered statistically significant.

## 3. Results

### 3.1. Baseline Characteristics and Outcomes

We enrolled 1670 patients with a mean age of 75 years (23–89 years). The demographics and clinical characteristics of the patients are summarized in Table 1. A total of 813 (49%) patients were categorized as NYHA functional class 3 and 857 as class 4. Among all patients, 586 (35%) patients were categorized as HFpEF and 1084 as HFrEF.

During the 12-month follow-up period after admission, all-cause death occurred in 360 patients, of which 294 experienced cardiovascular deaths. Cardiovascular deaths were caused by HF in 189 patients, myocardial infarction in 12, stroke in 17, sudden death in 57, and arrhythmia in 19.

Compared with survivors, the non-survivors were older and had a higher frequency of NYHA functional class 4; higher levels of GWTG-HF risk score, hs-CRP, NT-proBNP, hs-TnI, and D-dimer; and lower systolic blood pressure, hemoglobin levels, creatinine-based estimated glomerular filtration rate (eGFR), and LVEF. Many patients who experienced all-cause death had a previous myocardial infarction, prior hospitalization for worsening HF, mechanical ventilation before admission, intra-aortic balloon pump before admission, or use of diuretics. Patients who died also used statin or anticoagulant drugs less frequently than survivors. Among patients with AF, 318 (43%) were not on anticoagulant treatments at admission. The median length of CICU stay in non-survivors (3.0 (2.0–6.0) days) was longer than that in survivors (3.0 (2.0–4.0) days) (*p* < 0.001).

### 3.2. Prognostic Value of D-Dimer

**Figure 1:**
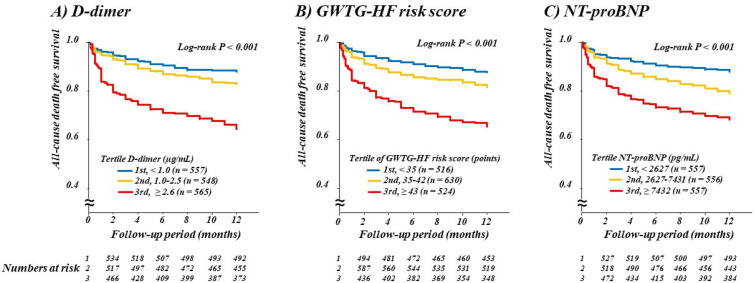
Kaplan-Meier curves for all-cause mortality according to tertiles of D-dimer, GWTG-HF risk score, and NT-proBNP. Numbers at risk in the respective groups are shown below the plots. GWTG-HF, Get With The Guidelines—Heart Failure; NT-proBNP, N-terminal pro-B-type natriuretic peptide. (**A**) D-dimer; (**B**) GWTG-HF risk score; (**C**) NT-proBNP.

**Figure 2:**
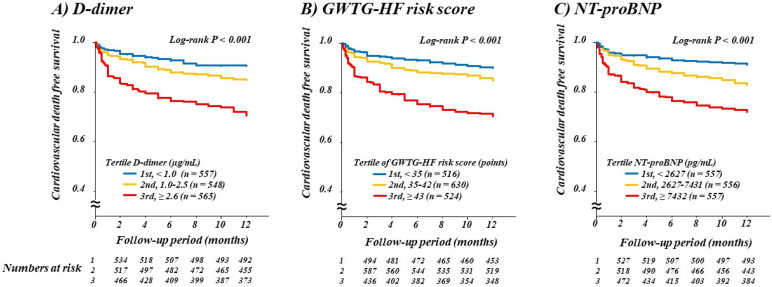
Kaplan-Meier curves for cardiovascular mortality according to tertiles of D-dimer, GWTG-HF risk score, and NT-proBNP. Numbers at risk of respective groups are shown below the plots. GWTG-HF, Get With The Guidelines—Heart Failure; NT-proBNP, N-terminal pro-B-type natriuretic peptide. (**A**) D-dimer; (**B**) GWTG-HF risk score; (**C**) NT-proBNP.

Patients were divided into tertiles according to D-dimer levels (1st, <1.0 µg/mL; 2nd, 1.0–2.5 µg/mL; and 3rd, ≥2.6 µg/mL), and Kaplan-Meier curves according to D-dimer tertiles revealed a graded increase in the risk of all-cause death (Figure 1; *p* for all <0.001) and cardiovascular death (Figure 2; *p* for all <0.001) with higher D-dimer levels. Similar results were obtained when patients were divided into tertiles according to GWTG-HF risk score (1st, <35 points; 2nd, 35–42 points; and 3rd, ≥43 points) and NT-proBNP (1st, <2627 pg/mL; 2nd, 2627–7431 pg/mL; and 3rd, ≥7432 pg/mL, all *p* for trend < 0.001; Figure 1 and Figure 2).

In the multivariate Cox analyses including all baseline variables with *p* < 0.05 by univariate analysis, D-dimer levels, GWTG-HF risk score, and NT-proBNP were independently associated with all-cause mortality when assessed as either continuous variables or variables categorized into two groups by the third tertile value in all patients, a subcohort of patients with HFpEF, and a subcohort of patients with HFrEF (all *p* < 0.001; Table 2 and Table 3). Similar results were obtained for cardiovascular mortality (Table 4 and Table 5).

### 3.3. Discrimination and Reclassification of D-Dimer for Mortality

We assessed the effect of adding D-dimer levels to a baseline model with the GWTG-HF risk score and NT-proBNP. Adding D-dimer levels improved the prediction of 12-month all-cause mortality beyond the baseline model alone in all populations, a subcohort of patients with HFpEF, and a subcohort of patients with HFrEF, as shown by NRI and IDI (all *p* < 0.001; Table 6). Similar results were obtained for cardiovascular mortality (Table 6).

### 3.4. Combination of D-Dimer with GWTG-HF Risk Score and NT-proBNP

**Figure 3:**
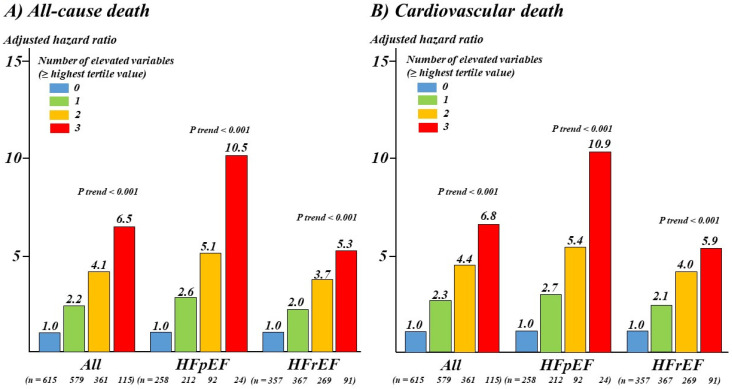
Adjusted hazard ratios of the number of elevations (≥the highest tertile value) in D-dimer levels (≥2.6 µg/mL), GWTG-HF risk score (≥43 points), and NT-proBNP (≥7432 pg/mL) for all-cause (**A**) and cardiovascular death (**B**) in all populations, HFpEF patients, and HFrEF patients. Multivariable models were adjusted for all baseline variables with *p* < 0.05 in univariate analyses. HFpEF—heart failure with preserved ejection fraction; HFrEF—heart failure with reduced ejection fraction.

Patients were divided according to the number of elevations (at least the highest tertile value) in D-dimer levels (≥2.6 µg/mL), GWTG-HF risk score (≥43 points), and NT-proBNP (≥7432 pg/mL). In the multivariate Cox analyses including all baseline variables with *p* < 0.05 by univariate analysis, adjusted relative risks of all-cause death for patients with elevation in all variables versus neither variable were approximately sevenfold higher in all populations, approximately 11-fold higher in a subcohort of HFpEF patients, and approximately five-fold higher in a subcohort of HFrEF (all *p* < 0.001; Figure 3). Similar results were obtained for cardiovascular mortality (Figure 3).

## 4. Discussion

The primary findings of this study were that plasma D-dimer levels upon admission, along with GWTG-HF risk score and serum NT-proBNP, are significant independent predictors of 12-month all-cause and cardiovascular mortality when considering all study populations, as well as HFpEF patients and HFrEF patients alone. Additionally, adding D-dimer levels to a baseline model with a GWTG-HF risk score and NT-proBNP improved the predictive value for 12-month all-cause and cardiovascular mortality among all study populations, HFpEF, and HFrEF patients, as demonstrated by the NRI and IDI. Finally, the number of elevations in D-dimer levels, GWTG-HF risk score, and NT-proBNP was independently correlated with an increased risk of 12-month all-cause and cardiovascular mortality graded fashion in all study populations, as well as HFpEF patients and HFrEF patients alone. These findings indicate that D-dimer levels on admission are a strong independent predictor of all-cause and cardiovascular mortality in acute decompensated HF patients with HFrEF or HFpEF. Moreover, the combined assessment of D-dimer levels with the GWTG-HF risk score and NT-proBNP levels may help the early risk stratification of patients hospitalized for worsening HF, regardless of the HF phenotype.

Previous investigations demonstrated the prognostic value of D-dimer levels mainly in patients with HFrEF [4,5,7]. In 1355 elderly patients admitted with chronic HF, Yan et al. [6] found an independent association between D-dimer levels and all-cause mortality in HFpEF and HFrEF patients. However, only about 36% of their study patients had NYHA functional class 3 or 4. Thus, we only focused on acute decompensated HF patients, of which all were present in NYHA 3 or 4, and demonstrated, for the first time, the independent association of D-dimer levels with all-cause and cardiovascular mortality in patients with acute decompensated HFpEF as well as those with HFrEF.

A single biomarker approach primarily reflects one pathophysiologic aspect. Therefore, its risk stratification and adverse outcome prediction in acute HF, a heterogeneous syndrome with various phenotypes, is often limited [25,26]. Accordingly, a combination of several predictors is expected to improve the accuracy of risk estimation [27,28,29]. To this end, Shiraishi et al. [21] performed a combined assessment of B-type natriuretic peptide levels and the GWTG-HF risk score, which may be helpful for predicting in-hospital mortality among patients hospitalized for acute HF. This investigation is the first to demonstrate that a combined assessment of D-dimer levels with a GWTG-HF risk score and NT-proBNP levels may improve early prediction of 12-month all-cause and cardiovascular mortality in patients with acute decompensated HF, regardless of the phenotype of HF. D-dimer, a marker of coagulation, is a valuable indicator of both coagulation and fibrinolysis [30,31], and provides different information than that provided by GWTG-HF risk score and NT-proBNP. Thus, the combined assessment of both the GWTG-HF risk score and NT-roBNP can be clinically beneficial. Each of these predictors is readily measured, easily accessible, and relatively inexpensive to measure. Therefore, a combined assessment of D-dimer levels with the GWTG-HF risk score and NT-proBNP is simple, has a robust discriminative capacity, and may help stratify 12-month all-cause and cardiovascular mortality risk in patients hospitalized for worsening HF, regardless of HF phenotype.

Recently, Zhao et al. demonstrated the predictive power of the plasma D-dimer/fibrinogen ratio in patients hospitalized for HF [32]. Further investigation is needed to evaluate the combination of D-dimer with other biomarkers. In addition, coagulopathy is a key feature of coronavirus disease (COVID-19). Recent studies reported that elevated D-dimer levels have consistently been shown as an important feature of severe COVID-19 patients [33].

This investigation study has several limitations. First, the study was conducted at a single institution and we only assessed D-dimer levels at the time of enrollment. Therefore, we did not evaluate whether D-dimer levels can function as a monitoring marker and whether improvements in this biomarker would impact the study outcome. In addition, atrial fibrillation (AF) is known to be associated with hemostatic abnormalities and often coexists with HF [34,35]. Our results suggest that a combined assessment of D-dimer with the GWTG-HF risk score and NT-proBNP could help stratify the risk of all-cause and cardiovascular death in both patients with and without AF, indicating that this combined assessment may be helpful regardless of the presence of AF. It is also worth mentioning that treatments were not randomized in the present study, so it is difficult to evaluate their effects on mortality. Thus, we did not evaluate drug treatments using Cox multivariate analyses. In addition to infrequent statin use and anticoagulant drugs use, patients who died used diuretics more frequently than survivors in the present study. Therefore, differences in medications may have potential confounding effects on our results. However, when we entered these medications into our Cox multivariate analyses, D-dimer, GWTG-HF risk score, and NT-proBNP were all still independent and significant predictors of all-cause and cardiovascular death. Consequently, we believe that the medications did not significantly affect our results. Some inherited or acquired thrombophilia could be clinically evident with arterial events. However, we could not measure markers for thrombophilia such as antiphospholipid antibodies, factor V Leiden, and prothrombin mutation; nor were we able to evaluate causes of worsening HF in detail. A total of 251 (15%) patients were accompanied by pneumonia or other infection, which may be associated with worsening HF. Finally, while current guidelines support a third ejection fraction-based group (HF with mid-range ejection fraction; HFmrEF) [22], we could not perform the analysis because of the small number of HFmrEF patients. Further investigations are required to clarify this issue.

## 5. Conclusions

Plasma D-dimer levels on admission are potent and independent predictors of 12-month all-cause and cardiovascular mortality in both acute decompensated HFpEF and HFrEF patients. When used in combination with the GWTG-HF risk score and NT-proBNP levels, D-dimer levels substantially improve the early risk stratification of acute decompensated HF patients, regardless of the phenotype of HF.

## Figures and Tables

**Table 1:** Baseline characteristics of survivors and non-survivors.

	All (*N* = 1670)	Non-Survivors (*N* = 360)	Survivors (*N* = 1310)	*p*-Value
Clinical data				
Age, years	75 ± 13	78 ± 13	74 ± 13	<0.001
Male, *n* (%)	955 (57)	222 (62)	733 (56)	0.05
Hypertension, *n* (%)	1146 (69)	222 (62)	924 (71)	0.001
Dyslipidemia, *n* (%)	679 (41)	123 (34)	556 (42)	0.007
Diabetes, *n* (%)	657 (39)	142 (39)	515 (39)	0.96
Current or ex-smoker, *n* (%)	471 (28)	92 (26)	379 (29)	0.21
Previous myocardial infarction, *n* (%)	438 (26)	119 (33)	319 (24)	<0.001
Prior hospitalization for worsening HF, *n* (%)	865 (52)	227 (63)	638 (49)	<0.001
Previous cerebral infarction, *n* (%)	342 (21)	81 (23)	261 (20)	0.28
Previous coronary revascularization, *n* (%)	423 (25)	104 (29)	319 (24)	0.08
Paroxysmal or persistent AF, *n* (%)	732 (44)	149 (41)	583 (45)	0.29
NYHA functional class 4, *n* (%)	857 (51)	224 (62)	633 (48)	<0.001
GWTG-HF risk score, point	38.8 ± 8.4	42.9 ± 8.5	37.6 ± 8.0	<0.001
Systolic BP, mmHg	137 ± 34	128 ± 34	139 ± 34	<0.001
Heart rate, beats per minutes	93 ± 28	94 ± 28	93 ± 28	0.83
Mechanical ventilation before admission, *n* (%)	70 (4.2)	34 (9.4)	36 (2.7)	<0.001
IABP before admission, *n* (%)	25 (1.5)	14 (3.9)	11 (0.8)	<0.001
Laboratory data				
WBC, ×10^3^/μL	7.9 ± 3.6	7.9 ± 4.2	7.9 ± 3.4	0.85
Hemoglobin, g/dL	11.6 ± 2.3	11.1 ± 2.1	11.8 ± 2.3	<0.001
eGFR, mL/min/1.73 m^2^	48.9 ± 25.3	43.3 ± 24.8	50.4 ± 25.2	<0.001
Glucose, mg/dL	149 ± 67	152 ± 68	148 ± 67	0.36
hs-CRP, mg/L	5.29 (1.58–20.7)	9.83 (3.00–36.8)	4.30 (1.40–16.7)	<0.001
NT-proBNP, pg/mL	4466 (2025–10,328)	7198 (3268–16,294)	3760 (1829–8801)	<0.001
hs-TnI, pg/mL	60 (21–181)	105 (42–340)	51 (17–154)	<0.001
D-dimer, µg/mL	1.6 (0.7–3.4)	2.9 (1.3–6.4)	1.3 (0.7–2.8)	<0.001
LVEF, %	41 ± 15	39 ± 15	41 ± 15	0.03
Medication at enrollment, n (%)				
RAAS inhibitors	812 (49)	165 (46)	647 (49)	0.23
Beta-blockers	856 (51)	193 (54)	663 (51)	0.31
Diuretics	716 (65)	173 (76)	543 (62)	<0.001
Statins	566 (34)	101 (28)	465 (36)	0.008
Antiplatelet drugs	456 (41)	105 (46)	351 (40)	0.09
Anticoagulant drugs	589 (35)	106 (29)	483 (37)	0.009

Data are expressed as number (%), mean ± standard deviation, or median (25th–75th percentile). HF, heart failure; AF, atrial fibrillation; NYHA, New York Heart Association; GWTG-HF, Get With The Guidelines—Heart Failure; BP, blood pressure; IABP, intra-aortic balloon pump; WBC, white blood cell; eGFR, creatinine-based estimated glomerular filtration rate; hs-CRP, high-sensitivity C-reactive protein; NT-proBNP, N-terminal pro-B-type natriuretic peptide; hs-TnI, high-sensitivity cardiac troponin I; LVEF, left ventricular ejection fraction; RAAS, renin-angiotensin-aldosterone system.

**Table 2:** Multivariate predictors of all-cause mortality in all study populations.

	Model 1			Model 2	
Variables	HR (95% CI)	*p*-Value	Variables	HR (95% CI)	*p*-Value
GWTG-HF risk score per 10 point increment	1.67 (1.45–1.92)	<0.001	GWTG-HF risk score <43 points (1st and 2nd tertile)	Ref	
			≥43 points (3rd tertile)	1.98 (1.58–2.50)	<0.001
NT-proBNP per 10-fold increment	1.54 (1.19–1.99)	0.001	NT-proBNP <7432 pg/mL (1st and 2nd tertile)	Ref	
			≥7432 pg/mL (3rd tertile)	1.41 (1.10–1.80)	0.007
D-dimer per 10-fold increment	2.61 (2.08–3.28)	<0.001	D-dimer <2.6 µg/mL (1st and 2nd tertile)	Ref	
			≥2.6 µg/mL (3rd tertile)	2.19 (1.77–2.72)	<0.001

The multivariable model was adjusted for all baseline variables with *p* < 0.05 in univariate analyses (i.e., hypertension, previous myocardial infarction, paroxysmal or persistent atrial fibrillation, prior hospitalization for worsening heat failure, New York Heart Association functional class, mechanical ventilation before admission, hemoglobin, creatinine-based estimated glomerular filtration rate, high-sensitivity C-reactive protein, high-sensitivity cardiac troponin I, and left ventricular ejection fraction). The GWTG-HF risk score, NT-proBNP, and D-dimer levels were assessed as either continuous variables (Model 1) or variables categorized into two groups by highest tertile value (Model 2). HR, hazard ratio; CI, confidence interval; GWTG-HF, Get With The Guidelines—Heart Failure; NT-proBNP, N-terminal pro-B-type natriuretic peptide.

**Table 3:** Multivariate predictors of all-cause mortality in HFpEF (A) and HFrEF (B) patients.

(A) HFpEF.					
	Model 1			Model 2	
Variables	HR (95% CI)	*p*-Value	Variables	HR (95% CI)	*p*-Value
GWTG-HF risk score per 10-point increment	1.59 (1.22–2.07)	<0.001	GWTG-HF risk score <43 points (1st and 2nd tertile)	Ref	
			≥43 points (3rd tertile)	2.36 (1.57–3.56)	<0.001
NT-proBNP per 10-fold increment	1.87 (1.23–2.84)	0.003	NT-proBNP <7432 pg/mL (1st and 2nd tertile)	Ref	
			≥7432 pg/mL (3rd tertile)	1.67 (1.04–2.69)	0.03
D-dimer per 10-fold increment	2.66 (1.77–4.00)	<0.001	D-dimer <2.6 µg/mL (1st and 2nd tertile)	Ref	
			≥2.6 µg/mL (3rd tertile)	2.38 (1.60–3.55)	<0.001
**(B) HFrEF.**					
GWTG-HF risk score per 10-point increment	1.68 (1.42–1.99)	<0.001	GWTG-HF risk score <43 points (1st and 2nd tertile)	Ref	
			≥43 points (3rd tertile)	1.81 (1.37–2.38)	<0.001
NT-proBNP per 10-fold increment	1.38 (1.01–1.88)	0.05	NT-proBNP <7432 pg/mL (1st and 2nd tertile)	Ref	
			≥7432 pg/mL (3rd tertile)	1.35 (1.02–1.79)	0.04
D-dimer per 10-fold increment	2.62 (1.99–3.46)	<0.001	D-dimer <2.6 µg/mL (1st and 2nd tertile)	Ref	
			≥2.6 µg/mL (3rd tertile)	2.14 (1.65–2.77)	<0.001

The multivariable model for HFpEF (A) and HFrEF (B) patients was adjusted for all baseline variables with *p* < 0.05 in univariate analyses (i.e., hypertension, previous myocardial infarction, paroxysmal or persistent atrial fibrillation, prior hospitalization for worsening heart failure, New York Heart Association functional class, mechanical ventilation before admission, hemoglobin, creatinine-based estimated glomerular filtration rate, high-sensitivity C-reactive protein, and high-sensitivity cardiac troponin I). The GWTG-HF risk score, NT-proBNP, and D-dimer levels were assessed as either continuous variables (Model 1) or variables categorized into two groups by highest tertile value (Model 2). HFpEF, heart failure with preserved ejection fraction; HFrEF, heart failure with reduced ejection fraction; HR, hazard ratio; CI, confidence interval; GWTG-HF, Get With The Guidelines—Heart Failure; NT-proBNP, N-terminal pro-B-type natriuretic peptide.

**Table 4:** Multivariate predictors of cardiovascular mortality in all study populations.

	Model 1			Model 2	
Variables	HR (95% CI)	*p*-Value	Variables	HR (95% CI)	*p*-Value
GWTG-HF risk score per 10-point increment	1.72 (1.46–2.01)	<0.001	GWTG-HF risk score <43 points (1st and 2nd tertile)	Ref	
			≥43 points (3rd tertile)	2.04 (1.58–2.64)	<0.001
NT-proBNP per 10-fold increment	1.62 (1.22–2.15)	<0.001	NT-proBNP <7432 pg/mL (1st and 2nd tertile)	Ref	
			≥7432 pg/mL (3rd tertile)	1.52 (1.16–1.99)	0.002
D-dimer per 10-fold increment	2.50 (1.94–3.21)	<0.001	D-dimer <2.6 µg/mL (1st and 2nd tertile)	Ref	
			≥2.6 µg/mL (3rd tertile)	2.09 (1.65–2.66)	<0.001

The multivariable model was adjusted for all baseline variables with *p* < 0.05 in univariate analyses (i.e., hypertension, previous myocardial infarction, paroxysmal or persistent atrial fibrillation, prior hospitalization for worsening heat failure, New York Heart Association functional class, mechanical ventilation before admission, hemoglobin, creatinine-based estimated glomerular filtration rate, high-sensitivity C-reactive protein, high-sensitivity cardiac troponin I, and left ventricular ejection fraction). The GWTG-HF risk score, NT-proBNP, and D-dimer levels were assessed as either continuous variables (Model 1) or variables categorized into two groups by highest tertile value (Model 2). HR, hazard ratio; CI, confidence interval; GWTG-HF, Get With The Guidelines—Heart Failure; NT-proBNP, N-terminal pro-B-type natriuretic peptide.

**Table 5:** Multivariate predictors of cardiovascular mortality in HFpEF (A) and HFrEF (B) patients.

(A) HFpEF.					
	Model 1			Model 2	
Variables	HR (95% CI)	*p*-Value	Variables	HR (95% CI)	*p*-Value
GWTG-HF risk score per 10 point increment	1.58 (1.17–2.12)	0.003	GWTG-HF risk score <43 points (1st and 2nd tertile)	Ref	
			≥43 points (3rd tertile)	2.44 (1.54–3.85)	<0.001
NT-proBNP per 10-fold increment	1.82 (1.14–2.92)	0.01	NT-proBNP <7432 pg/mL (1st and 2nd tertile)	Ref	
			≥7432 pg/mL (3rd tertile)	1.77 (1.04–2.99)	0.03
D-dimer per 10-fold increment	2.65 (1.67–4.19)	<0.001	D-dimer <2.6 µg/mL (1st and 2nd tertile)	Ref	
			≥2.6 µg/mL (3rd tertile)	2.30 (1.47–3.58)	<0.001
**(B) HFrEF.**					
GWTG-HF risk score per 10-point increment	1.76 (1.46–2.12)	<0.001	GWTG-HF risk score <43 points (1st and 2nd tertile)	Ref	
			≥43 points (3rd tertile)	1.91 (1.41–2.59)	<0.001
NT-proBNP per 10-fold increment	1.59 (1.12–2.24)	0.009	NT-proBNP <7432 pg/mL (1st and 2nd tertile)	Ref	
			≥7432 pg/mL (3rd tertile)	1.52 (1.12–2.08)	0.008
D-dimer per 10-fold increment	2.48 (1.83–3.37)	<0.001	D-dimer <2.6 µg/mL (1st and 2nd tertile)	Ref	
			≥2.6 µg/mL (3rd tertile)	2.03 (1.53–2.69)	<0.001

The multivariable model for HFpEF (A) and HFrEF (B) was adjusted for all baseline variables with *p* < 0.05 in univariate analyses (i.e., hypertension, previous myocardial infarction, paroxysmal or persistent atrial fibrillation, prior hospitalization for worsening heart failure, New York Heart Association functional class, mechanical ventilation before admission, hemoglobin, creatinine-based estimated glomerular filtration rate, high-sensitivity C-reactive protein, and high-sensitivity cardiac troponin I). The GWTG-HF risk score, NT-proBNP, and D-dimer levels were assessed as either continuous variables (Model 1) or variables categorized into two groups by highest tertile value (Model 2). HFpEF, heart failure with preserved ejection fraction; HFrEF, heart failure with reduced ejection fraction; HR, hazard ratio; CI, confidence interval; GWTG-HF, Get With The Guidelines—Heart Failure; NT-proBNP, N-terminal pro-B-type natriuretic peptide.

**Table 6:** Discrimination and reclassification of the combination of D-dimer with the GWTG-HF risk Score and NT-proBNP for all-cause and cardiovascular mortality in all populations (A), patients with HFpEF (B), and patients with HFrEF (C).

(A) All.						
All-cause Mortality						
	C-index	*p*-Value	NRI	*p*-Value	IDI	*p*-Value
Baseline model	0.704	Ref	0.326	Ref	0.021	Ref
Baseline model + D-dimer	0.747	0.05	0.575	<0.001	0.071	<0.001
**Cardiovascular Mortality**						
	**C-index**	***p*-Value**	**NRI**	***p*-Value**	**IDI**	***p*-Value**
Baseline model	0.702	Ref	0.312	Ref	0.022	Ref
Baseline model + D-dimer	0.740	0.10	0.542	<0.001	0.052	<0.001
**(B) HFpEF.**						
**All-cause Mortality**						
	**C-index**	***p*-Value**	**NRI**	***p*-Value**	**IDI**	***p*-Value**
Baseline model	0.694	Ref	0.360	Ref	0.033	Ref
Baseline model + D-dimer	0.752	0.06	0.599	<0.001	0.090	<0.001
**Cardiovascular Mortality**						
	**C-index**	***p*-Value**	**NRI**	***p*-Value**	**IDI**	***p*-Value**
Baseline model	0.694	Ref	0.368	Ref	0.026	Ref
Baseline model + D-dimer	0.751	0.06	0.573	<0.001	0.063	<0.001
**(C) HFrEF.**						
**All-cause Mortality**						
	**C-index**	***p*-Value**	**NRI**	***p*-Value**	**IDI**	***p*-Value**
Baseline model	0.700	Ref	0.274	Ref	0.015	Ref
Baseline model + D-dimer	0.742	0.11	0.547	<0.001	0.061	<0.001
**Cardiovascular Mortality**						
	**C-index**	***p*-Value**	**NRI**	***p*-Value**	**IDI**	***p*-Value**
Baseline model	0.697	Ref	0.282	Ref	0.018	Ref
Baseline model + D-dimer	0.731	0.21	0.522	<0.001	0.045	<0.001

Baseline models consist of GWTG-HF risk score and NT-proBNP. NRI, net reclassification improvement; IDI, integrated discrimination improvement; HFpEF, heart failure with preserved ejection fraction; HFrEF, heart failure with reduced ejection fraction; GWTG-HF, Get With The Guidelines—Heart Failure; NT-proBNP, N-terminal pro-B-type natriuretic peptide.

## Data Availability

The data presented in this study are available from the corresponding author on reasonable request.
